# Experimental and Numerical Study on Shear Resistance of Notched Perfobond Shear Connector

**DOI:** 10.3390/ma12030341

**Published:** 2019-01-22

**Authors:** Shuangjie Zheng, Yuqing Liu, Yangqing Liu, Chen Zhao

**Affiliations:** 1 College of Civil Engineering, Huaqiao University, Xiamen 361021, China; 2 Department of Bridge Engineering, Tongji University, Shanghai 200092, China; yql@tongji.edu.cn (Y.L.); 1432232@tongji.edu.cn (Y.L.); 3 Shanghai Municipal Engineering Design Institute (Group) Co., Ltd., Shanghai 200092, China; zhaochen@smedi.com

**Keywords:** composite structures, perfobond connector, shear capacity, push-out test, notched hole

## Abstract

In steel and concrete composite bridges, it is difficult to perforate the reinforcing bars through the circular holes of conventional perfobond shear connectors with multi-ribs. To ease the installation of perforating rebars, an alternative notched perfobond shear connector was proposed by cutting out the edge of the circular hole. This paper presents the push-out test results of six specimens which were fabricated and loaded to failure. The main purpose was to compare the failure mode, shear capacity and slip behavior of perfobond shear connectors using circular holes and notched holes. Furthermore, 43 nonlinear finite element simulations were performed to further study the effects of several variables, including the hole diameter, the hole distance, the hole number, the cut width, the perfobond thickness, the concrete strength, the rebar diameter, the rebar strength, and the steel strength. The parametric results were generated to evaluate the shear capacity equations for perfobond shear connectors. Finally, an analytical model was developed to estimate the shear capacity of notched perfobond shear connectors.

## 1. Introduction

Steel and concrete composite structures are increasingly used in bridge engineering to achieve a balance between structural performance and construction cost. Several types of innovative composite bridge structures have been proposed, such as hybrid girders [1], composite trusses [2], pile cap strengthening [3], and composite girders with corrugated steel webs [4]. The shear connection between steel and concrete is one of the most critical issues in the design of composite structures. Various types of shear connectors have been proposed to ensure the load transfer between steel and concrete components, such as headed studs [5], bolted connectors [6], perfobond connectors [7,8,9], pin shear connectors [10], and puzzle-shaped composite dowels [11]. The most popular shear connector in practice is the headed stud shear connector, which resists the shear force by the shank and prevents the separation by the anchorage head [5]. However, the headed studs have some disadvantages, such as the requirement for specific welding equipment on construction sites and fatigue problems of the weld collar under cyclic loading [12].

To ease installation and to improve fatigue performance, an alternative perfobond shear connector was proposed and used in a composite truss railway bridge [13]. The conventional perfobond shear connector is a flat steel plate having a certain number of circular holes. After concrete casting, dowels will form in these holes to resist shear forces and prevent separation between steel and concrete. The perfobond shear connector has some advantages over the headed studs, such as easier installation by fillet welding, no obvious fatigue problems, and higher shear stiffness and shear capacity [13,14,15]. Therefore, many types of composite bridge structures began to use the perfobond shear connectors to carry great dynamic loads [2,3].

**Figure 1:**
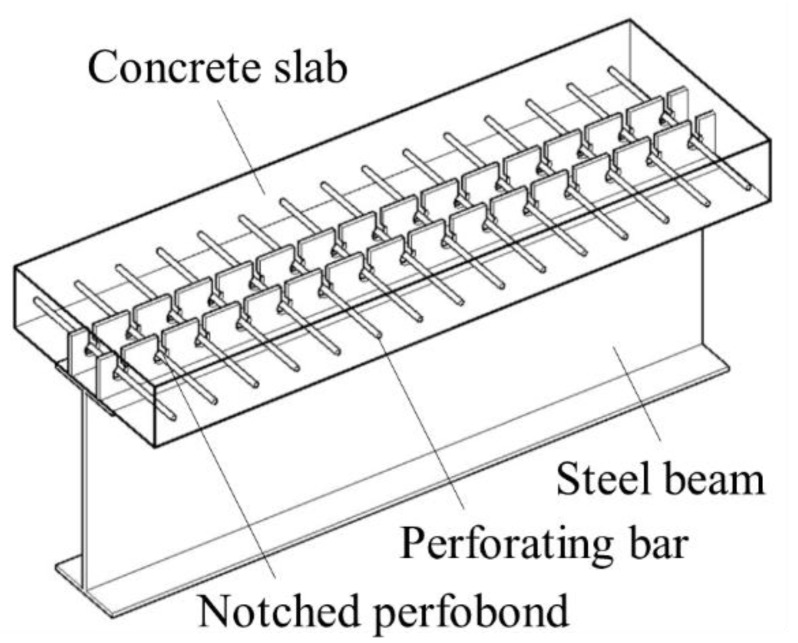
Proposed notched perfobond shear connector.

Several studies have been conducted to study the structural behavior of perfobond shear connectors mostly by push-out tests and finite element analysis. Leonhardt et al. [13] conducted push-out tests on perfobond shear connectors and proposed a shear capacity equation considering the effect of the concrete dowel. Oguejiofor et al. [14] completed experimental and numerical analysis to determine the contributions of bearing, concrete dowels, splitting resistance of the concrete and the transverse reinforcement. Hosaka et al. [15] performed several push-out tests and developed two equations for evaluating the shear capacity of perfobond shear connectors with and without rebar in the hole. Ahn et al. [16] proposed shear capacity equations of perfobond shear connectors considering the effects of concrete strength and rib arrangement. Zheng et al. [17] conducted parametric study of the shear capacity of long-hole perfobond shear connectors. Based on experimental and numerical analysis results, the shear stiffness and the shear capacity of perfobond shear connectors were greatly increased by providing a reinforcing bar through the holes [15,16,17]. However, when perfobond shear connectors are installed with multi-ribs parallel to each other, it is difficult to perforate the reinforcing bars through many holes of the ribs, which will reduce the construction efficiency. To solve this problem, a new type of notched perfobond shear connector was proposed by cutting out the edge of the circular hole of the conventional type, as shown in Figure 1. The reinforcing bar could be directly put into many holes at the same time, which will greatly speed the construction.

In this study, a total of six push-out tests were conducted to compare the failure modes and the load–slip behaviors of conventional and notched perfobond shear connectors. Moreover, 43 nonlinear finite element simulations were performed to further study the effects of several variables, including the hole diameter, the hole distance, the hole number, the cut width, the perfobond thickness, the concrete strength, the rebar diameter, the rebar strength, and the steel strength. The parametric results were generated to evaluate the shear capacity equations for perfobond shear connectors. Finally, an analytical model was developed to predict the shear capacity of notched perfobond shear connectors in steel and concrete composite bridges.

## 2. Experimental Investigations

### 2.1. Test Program

Table 1 presents six push-out tests on specimens with conventional perfobond and notched perfobond shear connectors, referring to the suggestions in Eurocode 4 [18]. The main variables of the push-out test specimens were the hole diameter *d_p_*, the cut width *c_w_*, the cut ratio *c_w_*/*d_p_*, the diameter of the rebar *d_r_*, the rib length *l_p_*, the rib height *h_p_*, and the rib thickness *t_p_*. The purpose was to verify the reliability of parametric study on push-out tests based on finite element analysis. As shown in Table 1, these specimens could be equally divided into two groups in terms of the connector type. The CPS specimens were conventional perfobond shear connectors with circular holes. The NPS specimens were notched perfobond shear connectors with cuts on the hole edge.

### 2.2. Layout of Test Specimen

**Figure 2:**
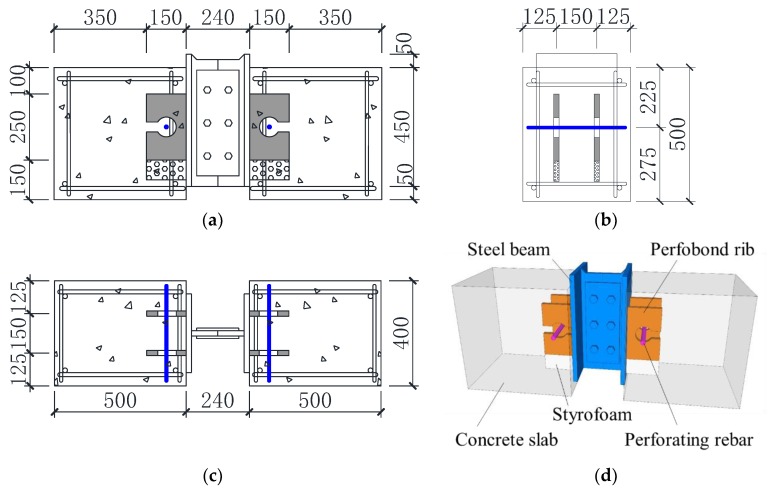
Layout of push-out test specimen (Units: mm): (**a**) front view; (**b**) side view; (**c**) top view; (**d**) 3D view.

The layout of a typical push-out test specimen is shown in Figure 2. All the test specimens were identical in terms of the dimensions except that CPS and NPS specimens had different configurations of perfobond ribs. Each specimen comprised one steel H-beam and two concrete slabs. The conventional perfobond and notched perfobond ribs were welded upright to the steel beam flange. A perforating rebar was fixed at the center of the hole for each perfobond rib. Bonding between steel and concrete was prevented by greasing the contact surfaces before concrete casting. Styrofoam was installed at the bottom of perfobond ribs to eliminate the bearing stress.

### 2.3. Details of Perfobond Ribs

**Figure 3:**
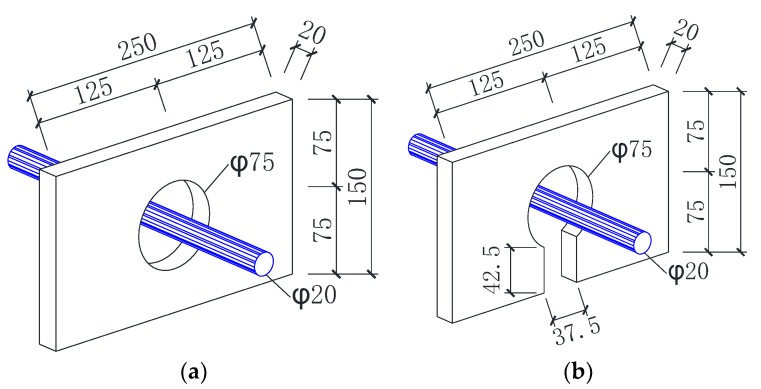
Details of perfobond ribs: (**a**) conventional perfobond rib; (**b**) notched perfobond rib.

As illustrated in Figure 3, the details of conventional perfobond rib and notched perfobond rib were identical in terms of the hole diameter (*d_p_* = 75 mm), the rib length (*l_p_* = 250 mm), the rib height (*h_p_* = 150 mm) and the rib thickness (*t_p_* = 20 mm). The conventional perfobond rib made a closed circular hole on the steel plate, while the notched perfobond rib made an open circular hole with a cut on the edge of the steel plate. In this test program, the cut width *c_w_* was designed as 37.5 mm, which was half of the hole diameter *d_p_*. Thus, the cut ratio *c_w_*/*d_p_* of the notched perfobond rib was equal to 0.5.

### 2.4. Material Properties

The concrete cube strength *f_cu_* was determined as 63.4 MPa from 150 mm × 150 mm × 150 mm concrete cube tests after a 28-day air curing period. The uniaxial compressive strength of concrete *f_c_* was 50.7 MPa which was equal to 0.8·*f_cu_*. The yield strength *f_ry_* and tensile strength *f_ru_* of rebar in the hole were 382.0 MPa and 547.0 MPa, respectively. The yield strength *f_sy_* and tensile strength *f_su_* of the structural steel were 410.0 MPa and 545.0 MPa, respectively.

### 2.5. Test Setup and Instrumentation

**Figure 4:**
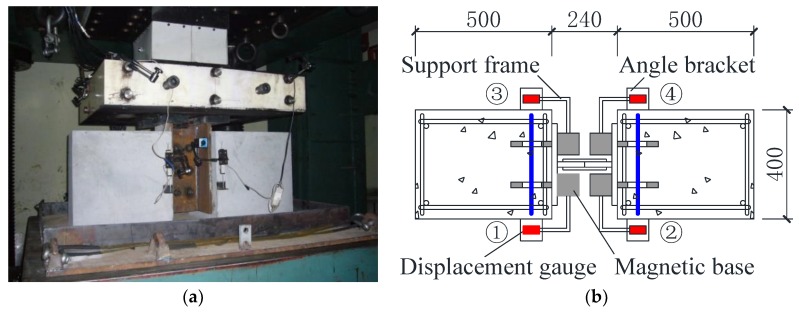
Test setup and instrumentation: (**a**) loading setup; (**b**) deformation measurements (Units: mm).

The push-out specimens were loaded to failure by using a hydraulic loading machine (Beijing Fluid Control System (FCS) Corp., Beijing, China) with a maximum capacity of 10,000 kN, as shown in Figure 4. The shear force between steel and concrete was applied by pushing down the steel H-beam. The first two specimens in each group were subjected to monotonic loading with displacement control. The loading rate was controlled to not reach the ultimate load in less than 15 min. The third specimen was loaded with uniaxial cyclic forces. The force control was adopted in the initial loading stage, followed by seven loading cycles with an increment of 10% of the tested shear load. The subsequent stage was a monotonic loading until complete failure. Four linear variable differential transformers (LVDTs) were symmetrically installed at the level of the perfobond shear connector to measure the relative slip between the steel beam and the concrete slab. The applied load and relative slips were continuously recorded. Therefore, the load–slip behaviors of conventional and notched perfobond shear connectors could be obtained to validate the proposed finite element model.

## 3. Finite Element Analysis

### 3.1. General

**Figure 5:**
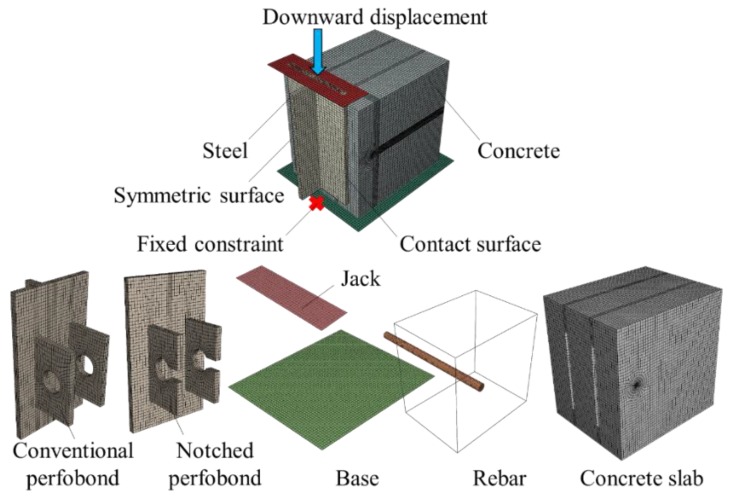
Finite element model.

As shown in Figure 5, the push-out tests of conventional and notched perfobond shear connectors were simulated by using the finite element method. Only one half of each specimen was built in finite element models to save analysis time. The main purpose of this analysis was to study the failure mechanism and the shear capacity by using validated finite element models instead of expensive and time-consuming push-out tests. The general analysis program ABAQUS (Version 6.10, Dassault System, Providence, RI, USA) [19] was adopted to simulate the push-out tests of conventional and notched perfobond shear connectors. The dynamic explicit method was adopted to consider both material and geometric nonlinearities. The loading rate was also carefully considered to assure quasi-static loading procedure.

### 3.2. Finite Element Type and Mesh

In this study, the push-out tests were modeled with symmetric constraints, as shown in Figure 5. Eight-node reduced integration elements (C3D8R) were chosen to model the concrete slab, the steel beam, the perfobond rib and the perforating rebar. Three-dimensional two-node truss elements (T3D2) were adopted to represent the other reinforcing bars embedded in concrete. Discrete rigid elements (R3D4) were used to mesh the jacking header and the base plate. In order to increase the accuracy of analysis, a locally refined mesh with a smallest size of about 5 mm was applied at the region near the notch and the hole of perfobond ribs. Global coarse mesh was applied with an overall size of 10 mm, 15 mm and 20 mm to save analysis time and clarify the sensitivity to mesh size.

### 3.3. Interaction and Boundary Conditions

The boundary condition (BC), as shown in Figure 5, was applied to the symmetric planes of the model. The reference point of the base plate was fixed in all directions. A downward enforced displacement was applied to the reference point of the jacking header. The perforating rebar was tied to the surrounding concrete in the hole. The other reinforcing bars were embedded inside the whole concrete slab. Contact interactions were applied at the interfaces of the concrete and shear connectors. A “hard” contact was used in the normal direction to prevent penetration, and the penalty frictional formulation was applied in the tangential direction. The frictional coefficient was taken as 0.5 for the contact between the base plate and the concrete slab, referring to previous research [17]. The other contact interactions were assumed to be frictionless.

### 3.4. Material Modeling of Concrete

**Figure 6:**
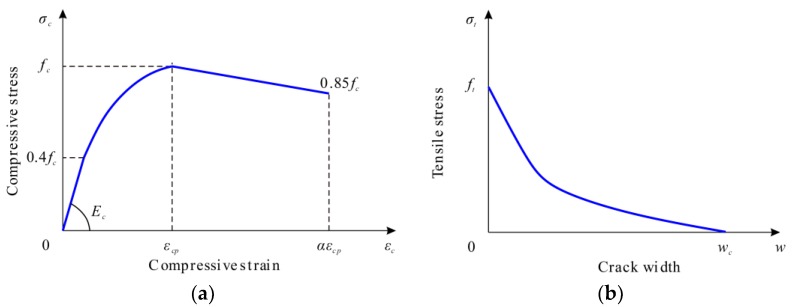
Material modeling of concrete: (**a**) compression; (**b**) tension.

As shown in Figure 6, the nonlinear behavior of the concrete material in compression and tension was represented by a uniaxial compressive stress–strain curve and a tensile stress–crack width relationship, respectively.

The concrete material constitutions in compression was governed by Equation (1) [20,21]. As shown in Figure 6a, the first branch of the stress–strain curve is assumed to be elastic. The following two branches are a nonlinear parabolic portion and a descending branch, respectively.

(1) $$\sigma_{c}=\{\begin{array}{ll} E_{c}\varepsilon_{c} & \left(0\leq \varepsilon_{c}\leq 0.4f_{c}/E_{c}\right)\\ \frac{k\cdot \eta -\eta^{2}}{1+\left(k-2\right)\cdot \eta }f_{c} & \left(0.4f_{c}/E_{c}<\varepsilon_{c}\leq \varepsilon_{cp}\right)\\ \left(1-0.15\frac{\varepsilon -\varepsilon_{cp}}{\varepsilon_{cu}-\varepsilon_{cp}}\right)f_{c} & \left(\varepsilon_{cp}<\varepsilon_{c}\leq \varepsilon_{cu}\right) \end{array}$$

where *σ_c_* is the compressive stress at any point (MPa); *ε_c_* is the compressive strain at any point; *E_c_* is Young’s modulus (MPa); *f_c_* is the compressive strength of concrete (MPa); *k* is the plasticity number, *k* = *E_c_*·*ε_cp_*/*f_c_*; *η* is the ratio of strain to peak strain, *η* = *ε_c_*/*ε_cp_*, *ε_cp_* = 0.002, and *ε_cu_* = 0.0033.

A linear stress–strain relationship was adopted to simulate uncracked concrete in tension. For a cracked section, as shown in Figure 6b, a nonlinear approach for the stress–crack width relationship can be determined by using Equation (2), referring to the study of Hordijk [22].

(2) $$\frac{\sigma_{t}}{f_{t}}=\left[1+{\left(c_{1}\cdot \frac{w}{w_{c}}\right)}^{3}\right]\cdot \exp\left(-c_{2}\cdot \frac{w}{w_{c}}\right)-\frac{w}{w_{c}}\cdot \left(1+c_{1}^{3}\right)\cdot \exp\left(-c_{2}\right)$$

where *σ_t_* is the tensile stress of concrete (MPa); *f_t_* is the tensile strength (MPa); *w* is the crack width (mm); *w_c_* is the crack width at the complete release of stress, *w_c_* = 5.14 *G_F_*/*f_t_* (mm); *G_F_* is the fracture energy required to create a unit area of stress-free crack, *G_F_* = 0.073 *f_c_*^0.18^ (N/mm); the constants are *c*_1_ = 3 and *c*_2_ = 6.93.

The concrete damaged plasticity model was adopted to depict the degraded response of the concrete material. Two independent uniaxial damage variables, *d_c_* and *d_t_*, were used to describe the damage of concrete due to compressive crushing and tensile cracking [19].

For concrete in compression, the evolution of *d_c_* is associated with the plastic strain *ε_c_^pl^*, determined proportional to the inelastic strain *ε_c_^in^* = (*ε_c_* − *σ_c_*)/*E_c_*, using a constant factor *b_c_* (0 < *b_c_* < 1) in Equation (3) as suggested in [23].

(3) $$d_{c}=1-\frac{\sigma_{c}}{E_{c}\cdot \varepsilon_{c}^{pl}\cdot \left(1/b_{c}-1\right)+\sigma_{c}}$$

where *d_c_* is the concrete compressive damage component; *b_c_* is the ratio of plastic strain to inelastic strain, *b_c_* = *ε_c_^pl^*/*ε_c_^in^*, and *b_c_* is taken as 0.7 [23].

For concrete in tension, the damage evolution component *d_t_* is related to the “plastic” crack width *w^pl^*, which is proportional to the crack width *w*, using a constant factor *b_t_* (0 < *b_t_* < 1) in Equation (4), referring to [23].

(4) $$d_{t}=1-\frac{\sigma_{t}\cdot l_{0}}{E_{c}\cdot w^{pl}\cdot \left(1/b_{t}-1\right)+\sigma_{t}\cdot l_{0}}$$

where *d_t_* is the tensile damage variable of concrete; *l*_0_ is assumed to be unit length; *b_t_* is the ratio of the “plastic” crack width to the crack width, *b_t_* = *w^pl^*/*w*, and *b_t_* is set as 0.1 [23].

### 3.5. Material Modeling of Steel

**Figure 7:**
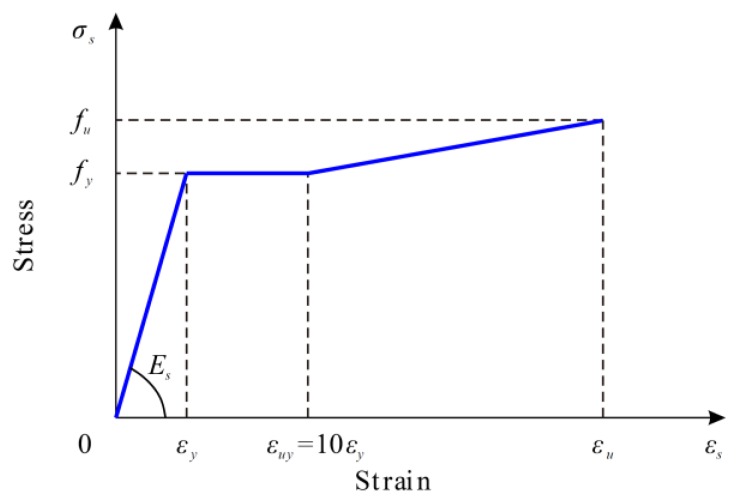
Material modeling of steel.

As shown in Figure 7, the stress–strain relationship of the structural and reinforcing steel was modeled by tri-linear curves. The initial regime is assumed to be elastic with Young’s modulus *E_s_*, followed by a stage of yielding and finally a branch of strain hardening. The stress–strain relationships for steel in tension and compression were assumed to be the same.

## 4. Analysis Results and Verification

### 4.1. Failure Mode

**Figure 8:**
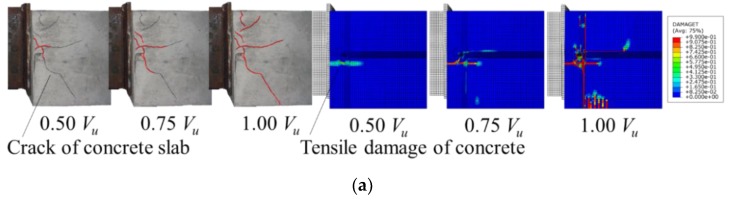

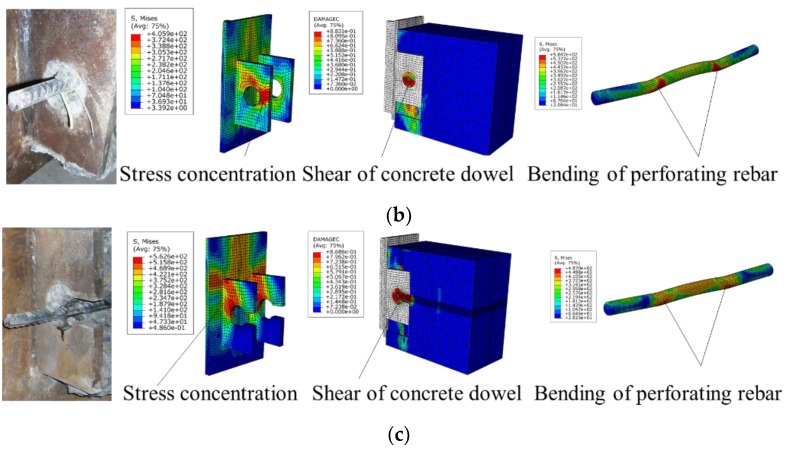
Tested and analyzed failure modes: (**a**) concrete slab; (**b**) conventional perfobond; (**c**) notched perfobond.

As shown in Figure 8, the numerical results resembled the push-out failures of conventional and notched perfobond shear connectors quite well. The failure modes were characterized by crack in the concrete slab, yield of the perforating rebar and shear failure of the concrete dowel. The concrete crack initially occurred near the perfobond shear connectors and spread out across the concrete slab as the load increased. The concrete slabs were demolished after specimen failure. The rebar in the hole yielded at the locations of perforation due to large shear and bending deformations. The concrete dowels in the hole failed in shear. There was no obvious deformation observed in the conventional perfobond rib. In comparison, the notched perfobond ribs were observed to deform as the cut width increased. Due to difficulties in simulating the nonlinear behavior of concrete materials, the analyzed failure modes were in reasonable agreement with the tested failure modes with a little discrepancy.

### 4.2. Load–Slip Behavior

**Figure 9:**
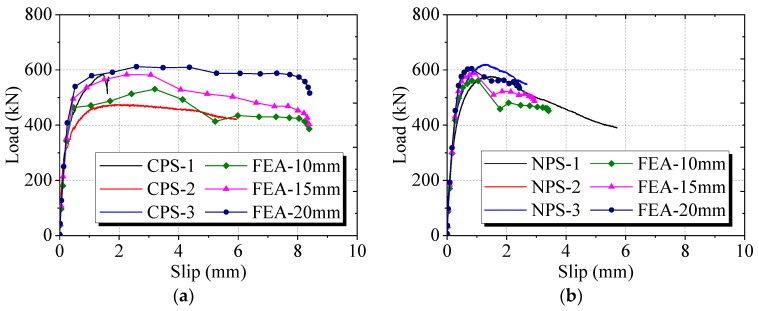
Tested and analyzed load–slip curves: (**a**) conventional perfobond shear connector; (**b**) notched perfobond shear connector.

As shown in Figure 9, the load–slip curves obtained from finite element analysis were in good agreement with push-out test results. Three stages were identified in the typical load–slip curves of both conventional and notched perfobond shear connectors. At the first stage, these curves were steep without obvious slips, indicating elastic behavior and large stiffness. The next stage was a nonlinear curve where the load increased and the stiffness reduced slowly with the slip. Beyond the peak load, the slip continued to increase as the load decreased. Before reaching the peak slip, the analyzed load-slip curves resembled the push-out test results quite well. However, it was difficult to predict the accurate post-failure behavior of notched perfobond shear connectors beyond the peak slip in the finite element analysis.

The accuracy of the proposed finite element model can be verified by comparison with push-out test results in Table 2. When the global meshing size were 10 mm, 15 mm and 20 mm, the analyzed shear capacities of conventional perfobond shear connectors accounted for 101%, 111% and 117% of the mean test results, while those of notched perfobond shear connectors accounted for 97%, 102% and 105% of the mean tested shear capacities. It was revealed that the proposed finite element model can be used to generate reasonable analysis results for both conventional and notched perfobond shear connectors when the overall element size was set as 10 mm.

### 4.3. Load Transfer Mechanism

**Figure 10:**
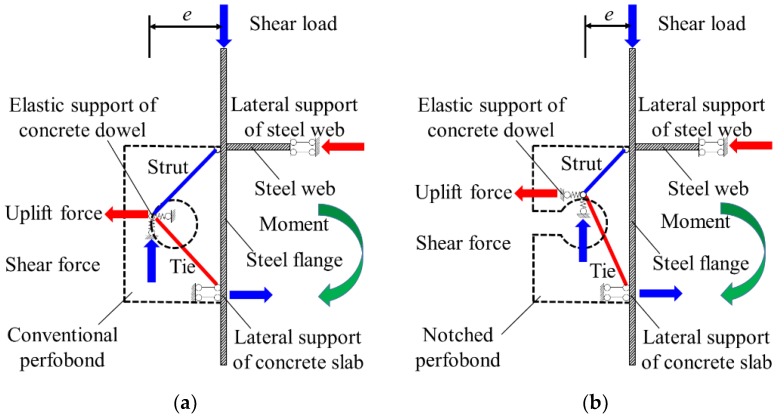
Load transfer mechanism: (**a**) conventional perfobond shear connector; (**b**) notched perfobond shear connector.

Based on the failure modes observed in push-out tests and numerical analysis, the load transfer mechanism of the conventional and notched perfobond shear connectors were compared in Figure 10. At first, the shear load was applied on the top of the steel beam flange and transmitted to the steel stem above the hole of perfobond ribs with an angle. Then the load was taken by the uplift and shear forces of the concrete dowel in lateral and longitudinal directions. Since there was an eccentricity between the shear load and the shear force of the concrete dowel, an additional bending moment occurred on the steel beam. This bending moment was balanced by the reaction forces provided by the lateral support of the steel web and the concrete slab. Finally, the shear force of the concrete dowel was further transferred to the perforating bar and the surrounding concrete. The conventional perfobond shear connector had a closed circular hole to resist the uplift force, which helped the concrete dowel to achieve full shear failure before the yield of the perfobond rib. In comparison, the notched perfobond shear connector made a cut on the hole edge, leading to smaller resistance to the uplift force. The yield of the perfobond rib occurred before the full shear failure of the concrete dowel. Therefore, the failure of the notched perfobond shear connector was characterized by shear of the concrete dowel, shear of the perforating rebar and yield of the perfobond rib.

## 5. Parametric Study

As shown in Table 3, a total of 43 push-out tests were simulated to study the further effects of connector dimension and material properties. The parameters included the hole diameter *d_p_*, the hole distance *e_p_*, the hole number *n_p_*, the cut width *c_w_*, the perfobond thickness *t_p_*, the concrete strength *f_cu_*, the rebar diameter *d_r_*, the yield strength *f_ry_* of the rebar, and the yield strength *f_sy_* of the structural steel.

### 5.1. Effect of Hole Diameter

**Figure 11:**
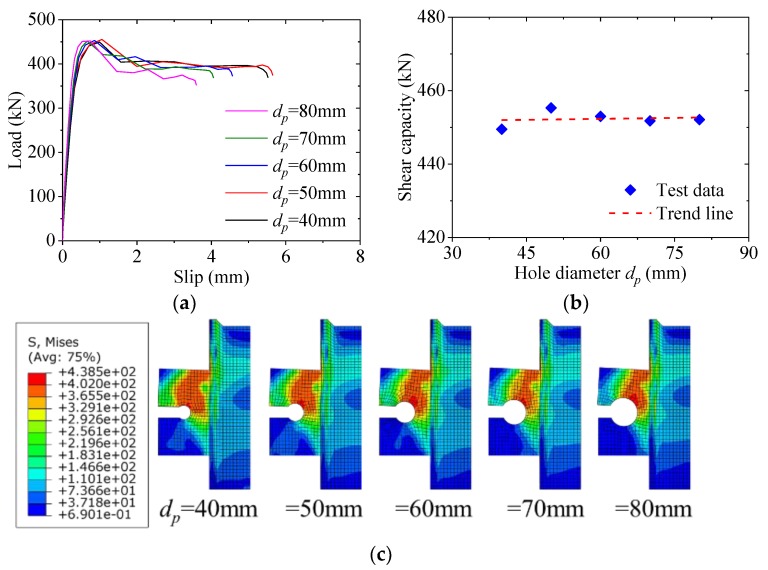
Effect of hole diameter: (**a**) load–slip curves; (**b**) influence analysis; (**c**) failure mode.

Figure 11 shows the effect of the hole diameter on the load–slip behavior of the notched perfobond shear connector. When the hole diameter was increased from 40 mm to 50 mm, 60 mm, 70 mm and 80 mm, the variations in the shear capacity were less than 2%. It was indicated that the increase of the hole diameter had little effect on the shear capacity of the notched perfobond shear connector. According to previous research, the increase of the hole diameter had great influence on the shear capacity of the conventional perfobond shear connector [13,14,15,16,17]. The main reason was that the failure mode of the conventional perfobond shear connector was directly related to the shear failure of the concrete dowel in the hole. When there was no cut, little deformation was observed in the perfobond rib, and the concrete dowel in the hole could reach full shear failure. In comparison, the notched perfobond shear connector had a cut on the hole edge, which resulted in failure of the perfobond rib before the concrete dowel could play its role. Therefore, the influence of the hole diameter between 40 mm and 80 mm on the shear capacity of the perfobond shear connector was negligible.

### 5.2. Effect of Hole Distance

**Figure 12:**
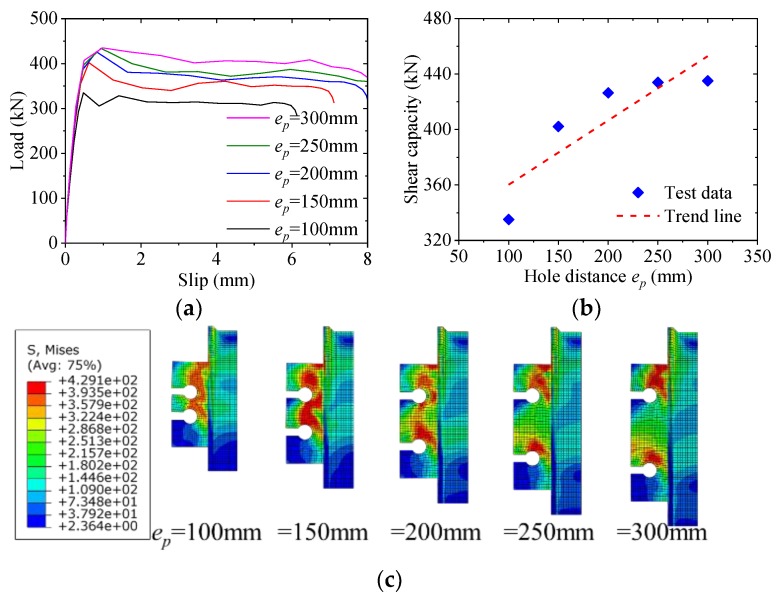
Effect of hole distance: (**a**) load–slip curves; (**b**) influence analysis; (**c**) failure mode.

Figure 12 shows the effect of the hole distance on the load–slip behavior of the notched perfobond shear connector. When the hole distance was increased from 100 mm to 150 mm, 200 mm, 250 mm and 300 mm, the average shear capacity increased by 20%, 27%, 30%, and 30%, respectively. This indicated that the increase of the hole distance led to increase in the average shear capacity of the notched perfobond shear connector. The reason might be that narrower hole distances resulted in smaller steel stems between the holes, which easily got fractured before the shear failure of the concrete dowel. When the hole distance was greater than 200 mm, the shear capacity continuously increased with lower amplitude. Therefore, the hole distance was suggested to be no smaller than 200 mm to prevent the steel fracture between the adjacent holes.

### 5.3. Effect of Hole Number

**Figure 13:**
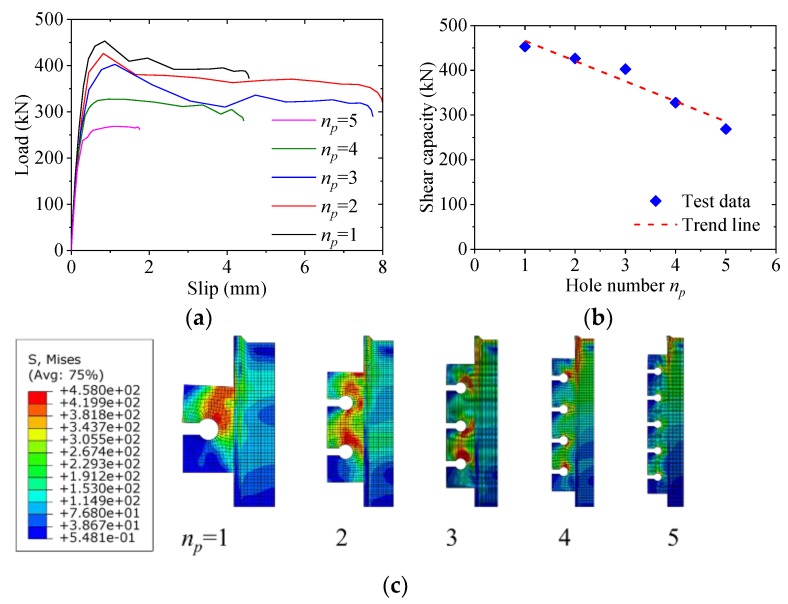
Effect of hole number: (**a**) load–slip curves; (**b**) influence analysis; (**c**) failure mode.

Figure 13 shows the effect of the hole number on the load–slip behavior of notched perfobond shear connector. When the hole number was increased from one to two, three, four and five, the average shear capacity per hole decreased by 6%, 11%, 28% and 41%, respectively. This indicated that the increase of the hole number had a significant effect on the average shear capacity of the notched perfobond shear connector. The main reason was that the shear load was unevenly distributed among the multi-holes in the longitudinal direction. The holes at the top and bottom of the perfobond rib bore greater loads than the holes at the middle of the perfobond rib. This was because the greatest slip deformation occurred at the top and at the bottom of the perfobond rib where the load and the reaction force applied. As a result, the average shear capacity of the multi-hole notched perfobond shear connector was smaller than the single-hole notched perfobond shear connector.

### 5.4. Effect of Cut Width

**Figure 14:**
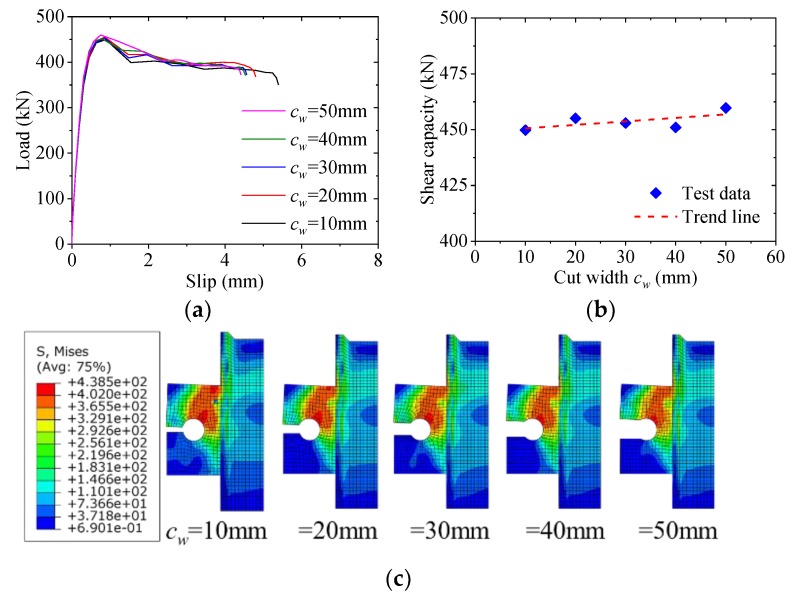
Effect of cut width. (**a**) Load–slip curves; (**b**) influence analysis; (**c**) failure mode.

Figure 14 shows the effect of the cut width on the load–slip behavior of the notched perfobond shear connector. When the cut width was increased from 10 mm to 20 mm, 30 mm, 40 mm and 50 mm, the differences of the shear capacities were less than 3%. This indicated that the increase of the cut width had a negligible effect on the shear capacity of the notched perfobond shear connector. The failure mode of the notched perfobond shear connector was directly related to the shear failure of the steel perfobond rib. Stress concentration could be observed at the hole edge of the perfobond shear connector due to contact interaction between the steel stem and the concrete dowel in the hole. This contact area was not affected by changing the cut width of the notched perfobond rib. As a result, the increase of the cut width had little influence on the shear capacity of the notched perfobond shear connector.

### 5.5. Effect of Perfobond Thickness

**Figure 15:**
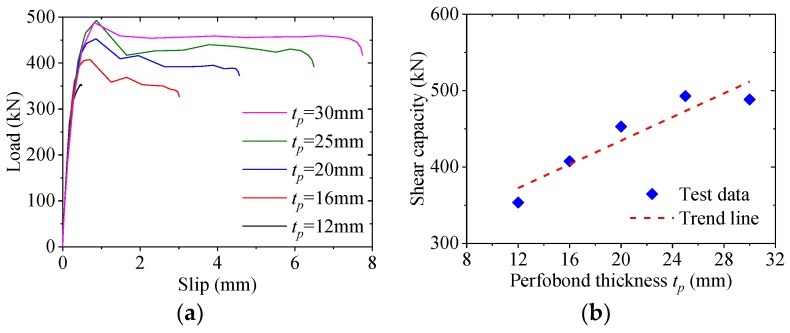

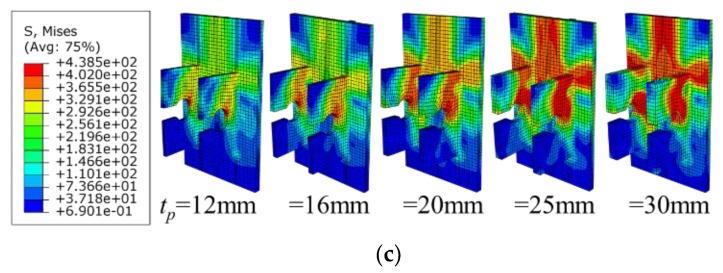
Effect of perfobond thickness. (**a**) Load–slip curves; (**b**) influence analysis; (**c**) failure mode.

Figure 15 shows the effect of the perfobond thickness on the load–slip behavior of the notched perfobond shear connector. When the thickness of the perfobond rib was increased from 12 mm to 16 mm, 20 mm, 25 mm and 30 mm, the shear capacity increased by 15%, 28%, 39% and 38%, respectively. This indicated that the increase of the perfobond thickness would lead to an increase in the shear capacity of the notched perfobond shear connector. The main reason was that increasing the thickness of the perfobond rib increased the cross-sectional area of the steel stem, which resulted in greater shear capacity of the notched perfobond shear connector.

### 5.6. Effect of Concrete Strength

**Figure 16:**
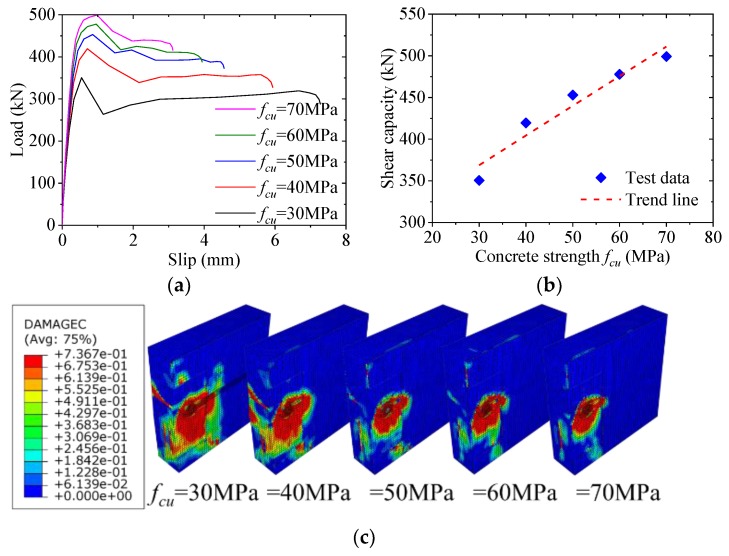
Effect of concrete strength: (**a**) load–slip curves; (**b**) influence analysis; (**c**) failure mode.

Figure 16 shows the effect of the concrete strength on the load–slip behavior of notched perfobond shear connector. When the concrete strength was increased from 30 MPa to 40 MPa, 50 MPa, 60 MPa and 70 MPa, the shear capacity increased by 20%, 29%, 36% and 43%, respectively. This indicated that the increase of the concrete strength would lead to increase in the shear capacity of the notched perfobond shear connector. The reason might be that when higher strength concrete was used, a smaller region of the damaged concrete below the concrete dowel was observed in the analyzed failure modes. As a result, the steel stem of the notched perfobond shear connector could reach full shear strength before the shear failure of the concrete dowel in the hole.

### 5.7. Effect of Rebar Diameter

**Figure 17:**
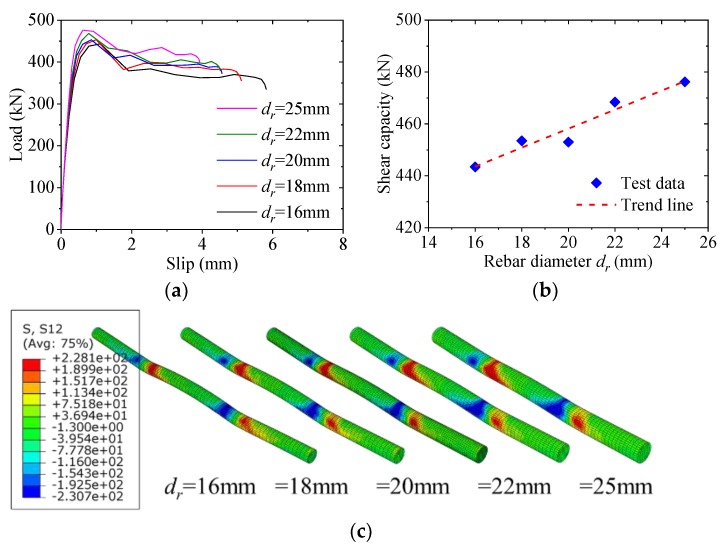
Effect of rebar diameter: (**a**) load–slip curves; (**b**) influence analysis; (**c**) failure mode.

Figure 17 shows the effect of the rebar diameter on the load–slip behavior of the notched perfobond shear connector. When the rebar diameter was increased from 16 mm to 18 mm, 20 mm, 22 mm and 25 mm, the shear capacity increased by 2%, 2%, 6% and 7%, respectively. This indicated that the increase of the rebar diameter would lead to increase in the shear capacity of the notched perfobond shear connector. When a larger rebar was used, a larger region of the concrete near the concrete dowel was involved in resisting the shear load, and less bending deformation was observed for the perforating rebar in the hole.

### 5.8. Effect of Rebar Strength

**Figure 18:**
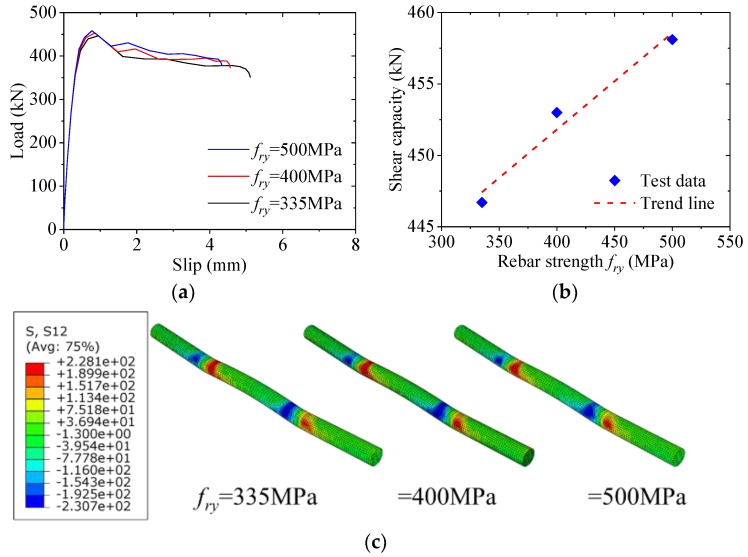
Effect of rebar strength: (**a**) load–slip curves; (**b**) influence analysis; (**c**) failure mode.

Figure 18 shows the effect of the rebar strength on the load–slip behavior of the notched perfobond shear connector. When the yield strength of the rebar was increased from 335 MPa to 400 MPa and to 500 MPa, the shear capacity increased by 1% and 3%, respectively. This indicated that the increase of the rebar strength would lead to increase in the shear capacity of the notched perfobond shear connector. The main reason was that when a higher strength rebar was used in the hole, the shear resistance of the rebar and that of the concrete dowel were both increased.

### 5.9. Effect of Steel Strength

**Figure 19:**
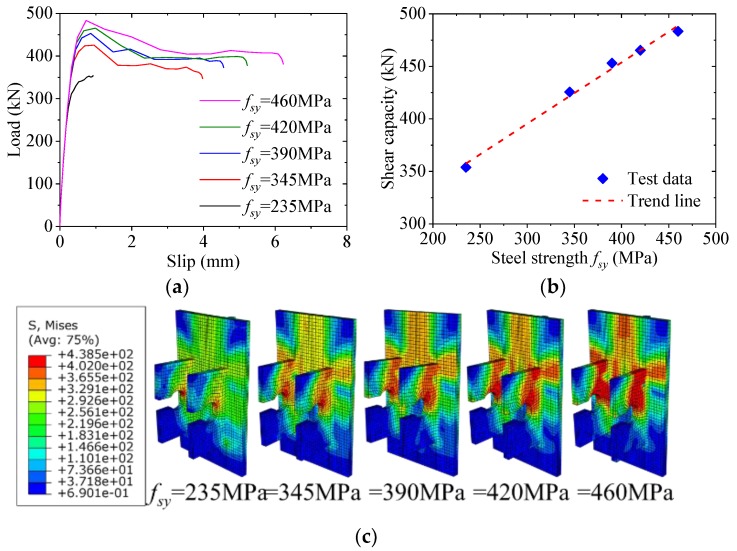
Effect of steel strength: (**a**) load–slip curves; (**b**) influence analysis; (**c**) failure mode.

As shown in Figure 19, when the yield strength of the perfobond rib was increased from 235 MPa to 345 MPa, 390 MPa, 420 MPa and 460 MPa, the shear capacity increased by 20%, 28%, 32% and 37%, respectively. This indicated that the increase of the steel strength would lead to great increase in the shear capacity of the notched perfobond shear connector. The reason was that when higher strength steel was used for the perfobond rib, the shear strength of the steel stem above the hole was increased, which resulted in greater shear capacity of the notched perfobond shear connector.

## 6. Prediction of Shear Capacity

### 6.1. Previous Expressions

In this study, bonding between steel and concrete was eliminated by greasing the steel surface, and the concrete-end bearing stress below the perfobond rib was prevented by installing Styrofoam. The shear capacity equations having similar conditions were chosen to evaluate the push-out test results.

Based on push-out test results, Leonhardt et al. [13] proposed Equation (5) to calculate the shear capacity of conventional perfobond shear connectors without considering the contribution of the rebar in the hole.

(5) $$V_{pu}=1.4d_{p}^{2}f_{cu}$$

where *V_pu_* is the shear capacity per hole (N); *d_p_* is the hole diameter (mm); *f_cu_* is the concrete cube strength (MPa).

Hosaka et al. [15] suggested Equation (6) to predict the shear capacity per hole of conventional perfobond shear connectors. Two different formulas were adopted to consider the influence of the perforating rebar.

(6) $$V_{pu}=\{\begin{array}{ll} 3.38\sqrt{t_{p}/d_{p}}\cdot d_{p}^{2}f_{c}-39.0\times 10^{3} & \mathrm{no}\;\mathrm{rebar}\;\mathrm{in}\;\mathrm{hole}\\ 1.45\left[\left(d_{p}^{2}-d_{r}^{2}\right)f_{c}+d_{r}^{2}f_{ru}\right]-26.1\times 10^{3} & \mathrm{rebar}\;\mathrm{in}\;\mathrm{hole} \end{array}$$

where *t_p_* is the thickness of the perfobond rib; *f_c_* is the concrete compressive strength (MPa); *d_r_* is the diameter of the rebar in the hole (mm); and *f_ru_* is the ultimate tensile strength of rebar (MPa).

### 6.2. Proposed Expression

Based on the experimental and numerical results, the notched perfobond shear connector failed due to shear of the concrete dowel, shear of the perforating rebar and yield of the steel stem. It was revealed by the parametric study that the contribution of the steel stem was related to the hole diameter, the perfobond thickness and the strength of steel. Therefore, an alternative equation, Equation (7), was proposed for evaluating the shear capacity per hole of the notched perfobond shear connector by combining these contributions.

(7) $$V_{pu}=\gamma_{n}\gamma_{e}\left[C_{1}\left(d_{p}^{2}-d_{r}^{2}\right)f_{c}+C_{2}d_{r}^{2}f_{ry}+C_{3}d_{p}t_{p}f_{sy}\right]$$

where *V_pu_* is the shear capacity per hole (N); *d_p_* is the hole diameter (mm); *d_r_* is the diameter of the rebar in the hole (mm); *f_c_* is the concrete compressive strength (MPa); *f_ry_* is the yield strength of rebar (MPa); *t_p_* is the perfobond thickness (mm); *f_sy_* is the yield strength of steel (MPa); *γ_n_* and *γ_e_* are coefficients considering the effects of the hole number and the hole distance, respectively; and *C*_1_, *C*_2_ and *C*_3_ are fitting coefficients.

Nonlinear regression analysis was conducted on a total of 46 experimental and numerical results; the best fitting of the coefficients in Equation (7) were derived as *C*_1_ = 0.42, *C*_2_ = 1.15, and *C*_3_ = 0.45. Therefore, the final proposal for estimating the shear capacity of notched perfobond shear connector could be given as Equation (8).

(8) $$\begin{array}{c} V_{pu}=\gamma_{n}\gamma_{e}\left[0.42\left(d_{p}^{2}-d_{r}^{2}\right)f_{c}+1.15d_{r}^{2}f_{ry}+0.45d_{p}t_{p}f_{sy}\right]\\ \;\mathrm{with}\;\gamma_{n}=n_{p}^{-0.22};\gamma_{e}=1+0.002\left(e_{p}-200\right)\leq 1 \end{array}$$

### 6.3. Comparison and Validation

**Figure 20:**
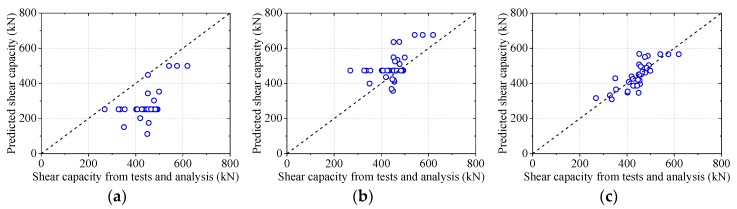
Comparison of shear capacity equations: (**a**) Leonhardt et al.; (**b**) Hosaka et al.; (**c**) proposal.

The calculated shear capacities from Equation (8) were compared to the experimental and numerical results. As shown in Figure 20, the equations suggested by Leonhardt et al. [13] underestimated the shear capacity of perfobond shear connectors by not considering the effect of the perforating rebar and the steel stem in Equation (5). The equation of Hosaka et al. [15] overestimated the shear capacity of notched perfobond shear connectors. The possible reason was that the tensile strength *f_ru_* was used in Equation (6) to calculate the contribution of rebar in the hole, while yielding of the rebar was observed at specimen failure instead of fracture. The predicted shear capacities from the proposal in this study agreed reasonably well with the results from push-out tests and parametric studies. Therefore, Equation (8) could be used to predict the shear capacity of notched perfobond shear connectors in steel and concrete composite structures.

## 7. Conclusions

In this paper, a new type of notched perfobond shear connector was proposed to ease the installation of perforating rebar through the holes. Based on experimental investigation, parametric analysis and analytical work, the following conclusions can be drawn:

(1) The push-out failure modes of perfobond shear connectors with circular and notched holes are characterized by crack in the concrete slab, yield of the perforating rebar and shear failure of the concrete dowel. The concrete crack initially occurs near the perfobond shear connectors and spreads out across the concrete slab. The rebar in the hole yields at the locations of perforation due to large deformations. The concrete dowels in the hole fails in shear. There is no obvious deformation in the conventional perfobond rib. In comparison, the notched perfobond rib deforms with the cut width increased.
(2) The accuracy of the proposed finite element method is verified by comparison with push-out test results. When the global mesh sizes were 10 mm, 15 mm and 20 mm, the analyzed shear capacities of conventional perfobond shear connectors accounted for 101%, 111% and 117% of the mean test results, respectively, while those of notched perfobond shear connectors took up 97%, 102% and 105% of the mean tested shear capacities. The proposed finite element model can be used to generate reasonable analysis results for both conventional and notched perfobond shear connectors with the overall element size of 10 mm.
(3) In total, 43 finite element models of push-out tests were generated to evaluate the shear capacity of notched perfobond shear connectors. The shear capacity could be increased by 38% by increasing the perfobond thickness from 12 mm to 30 mm, by 43% by increasing the concrete strength from 30 MPa to 70 MPa, by 37% by increasing the steel strength from 235 MPa to 460 MPa. The hole diameter, the cut width, the rebar diameter and the rebar strength have little effect on the shear capacity of notched perfobond shear connectors.
(4) Based on nonlinear finite element simulations of notched perfobond shear connectors, increasing the hole distance from 100 mm to 300 mm leads to an increase in the average shear capacity by 30%. The reason might be that narrower hole distances result in smaller steel stems between the holes which easily get fractured before the shear failure of the concrete dowel. The hole distance is suggested to be no smaller than 200 mm to prevent the steel fracture between the adjacent holes.
(5) The increase of the hole number from one to five leads to a decrease in the average shear capacity of the notched perfobond shear connector by 41%. The main reason is that the shear loads are unevenly distributed among the multi-holes in the longitudinal direction. The holes at two ends of the perfobond rib bear greater loads than the holes at the middle. The average shear capacity of the multi-hole notched perfobond shear connector is smaller than that of the single-hole notched perfobond shear connector.
(6) An analytical model is proposed to evaluate the shear capacity per hole of notched perfobond shear connectors. Compared with the existing equations, the predicted shear capacities from the proposal in this study agree reasonably well with the experimental and numerical results, and thus may be used to estimate the shear capacity of notched perfobond shear connectors. Further experimental studies are required to extend the scope of the proposed shear capacity equation in steel and concrete composite structures.

## Figures and Tables

**Table 1:** Push-out test specimens.

Specimen	*d_p_* (mm)	*c_w_* (mm)	*c_w_/d_p_*	*d_r_* (mm)	*l_p_* (mm)	*h_p_* (mm)	*t_p_* (mm)	Notch
CPS–1	75	-	0	20	250	150	20	No
CPS–2	75	-	0	20	250	150	20	No
CPS–3	75	-	0	20	250	150	20	No
NPS–1	75	37.5	0.5	20	250	150	20	Yes
NPS–2	75	37.5	0.5	20	250	150	20	Yes
NPS–3	75	37.5	0.5	20	250	150	20	Yes

**Table 2:** Tested and analyzed shear capacities.

Specimen.	Test		Analysis			Comparison		
	*V_u_*_,*i*_ (kN)	*V_u_*_,*avg*_ (kN)	*V_u_*_,*fea1*_ (kN)	*V_u_*_,*fea2*_ (kN)	*V_u_*_,*fea3*_ (kN)	*V_u_*_,*fea1*_/*V_u_*_,*avg*_	*V_u_*_,*fea2*_/*V_u_*_,*avg*_	*V_u_*_,*fea3*_/*V_u_*_,*avg*_
CPS–1	583.4							
CPS–2	474.9	524.6	530.5	582.9	611.7	101%	111%	117%
CPS–3	515.4							
NPS–1	575.4							
NPS–2	541.5	578.7	561.3	587.6	604.5	97%	102%	105%
NPS–3	619.2							

**Table 3:** Generalization of parametric models.

Model	*d_p_* (mm)	*e_p_* (mm)	*n_p_*	*c_w_* (mm)	*t_p_* (mm)	*f_cu_* (MPa)	*d_r_* (mm)	*f_ry_* (MPa)	*f_sy_* (MPa)	*V_u_* (kN)
DP–40	40	—	1	30	20	50	20	400	390	449.5
DP–50	50	—	1	30	20	50	20	400	390	455.3
DP–60	60	—	1	30	20	50	20	400	390	453.0
DP–70	70	—	1	30	20	50	20	400	390	451.8
DP–80	80	—	1	30	20	50	20	400	390	452.1
EP–100	60	100	2	30	20	50	20	400	390	335.1
EP–150	60	150	2	30	20	50	20	400	390	402.2
EP–200	60	200	2	30	20	50	20	400	390	426.4
EP–250	60	250	2	30	20	50	20	400	390	434.0
EP–300	60	300	2	30	20	50	20	400	390	435.1
NP–1	60	—	1	30	20	50	20	400	390	453.0
NP–2	60	200	2	30	20	50	20	400	390	426.4
NP–3	60	200	3	30	20	50	20	400	390	402.8
NP–4	60	200	4	30	20	50	20	400	390	327.5
NP–5	60	200	5	30	20	50	20	400	390	268.8
CW–10	60	—	1	10	20	50	20	400	390	449.8
CW–20	60	—	1	20	20	50	20	400	390	455.1
CW–30	60	—	1	30	20	50	20	400	390	453.0
CW–40	60	—	1	40	20	50	20	400	390	451.0
CW–50	60	—	1	50	20	50	20	400	390	459.7
TP–12	60	—	1	30	12	50	20	400	390	353.6
TP–16	60	—	1	30	16	50	20	400	390	407.6
TP–20	60	—	1	30	20	50	20	400	390	453.0
TP–25	60	—	1	30	25	50	20	400	390	493.0
TP–30	60	—	1	30	30	50	20	400	390	488.5
CU–30	60	—	1	30	20	30	20	400	390	350.3
CU–40	60	—	1	30	20	40	20	400	390	419.5
CU–50	60	—	1	30	20	50	20	400	390	453.0
CU–60	60	—	1	30	20	60	20	400	390	477.8
CU–70	60	—	1	30	20	70	20	400	390	499.2
DR–16	60	—	1	30	20	50	16	400	390	443.4
DR–18	60	—	1	30	20	50	18	400	390	453.5
DR–20	60	—	1	30	20	50	20	400	390	453.0
DR–22	60	—	1	30	20	50	22	400	390	468.4
DR–25	60	—	1	30	20	50	25	400	390	476.2
RY–335	60	—	1	30	20	50	20	335	390	446.7
RY–400	60	—	1	30	20	50	20	400	390	453.0
RY–500	60	—	1	30	20	50	20	500	390	458.1
SY–235	60	—	1	30	20	50	20	400	235	353.8
SY–345	60	—	1	30	20	50	20	400	345	425.6
SY–390	60	—	1	30	20	50	20	400	390	453.0
SY–420	60	—	1	30	20	50	20	400	420	465.4
SY–460	60	—	1	30	20	50	20	400	460	483.4

Note: DP is the hole diameter; EP is the hole distance; NP is the hole number; CW is the cut width; TP is the perfobond thickness; CU is the concrete strength; DR is the rebar diameter; RY is the yield strength of rebar; SY is the yield strength of steel.
